# Ten simple rules for designing your career pivot

**DOI:** 10.1371/journal.pcbi.1014890

**Published:** 2026-10-27

**Authors:** Amber R. Moore, Vinita Bharat

**Affiliations:** 1 Grant Writing Academy, School of Medicine, Stanford University, Stanford, California, United States of America; 2 Department of Pediatrics, School of Medicine, Stanford University, Stanford, California United States of America; Dassault Systemes BIOVIA Ltd, UNITED STATES OF AMERICA

## Introduction

We are navigating an unprecedented and highly volatile period in academia marked by research funding cuts, off-limits research topics, and tightening job markets, impacting scientists at all career stages [1,2]. While some scientists have fortified their resolve in pursuing an academic research career, for others, this experience may have inspired them to reevaluate their interests in the academic career path while seeking ways to contribute that better align with their values and priorities [2–4]. Amidst the chaos, fears, and uncertainty that career transitions might introduce (i.e., economic, social, etc.), the current state of precarity in academia might serve as a unique opportunity for change and an impetus for the latter to make a career pivot.

Several 10 Simple Rules articles offer comprehensive advice on choosing between government, industry, and academia, approaching a new job, transitioning from basic to public health research, or even finding the right job after completing a PhD or postdoctoral position [5–9]. However, there is a need for an article on pivoting careers at any stage and doing so in more creative and potentially joyful ways.

We present ten simple, actionable rules for career pivots inspired by creative applications of design thinking-a human-centered approach rooted in understanding a problem, iteration, and implementation-to solve life’s challenges [10–12] (Fig 1). The career pivot is yet another one of life’s challenges that can be approached creatively through design thinking, but also sustainably when emotional awareness and well-being are centered as they are here. The rules are offered in a logical sequence. However, career pivots are iterative, and you are encouraged to revisit previous rules as new insights emerge or jump ahead if that better serves you in the moment. The approach reflects our experiences as STEM postdocs pivoting into our current roles in research development. Throughout our career pivots, empathy and community enabled us to enjoy the process, as well as the result. We both found roles we didn’t previously know existed in academia, but that continue to bring us joy and align with our values and priorities.

**Fig 1:**
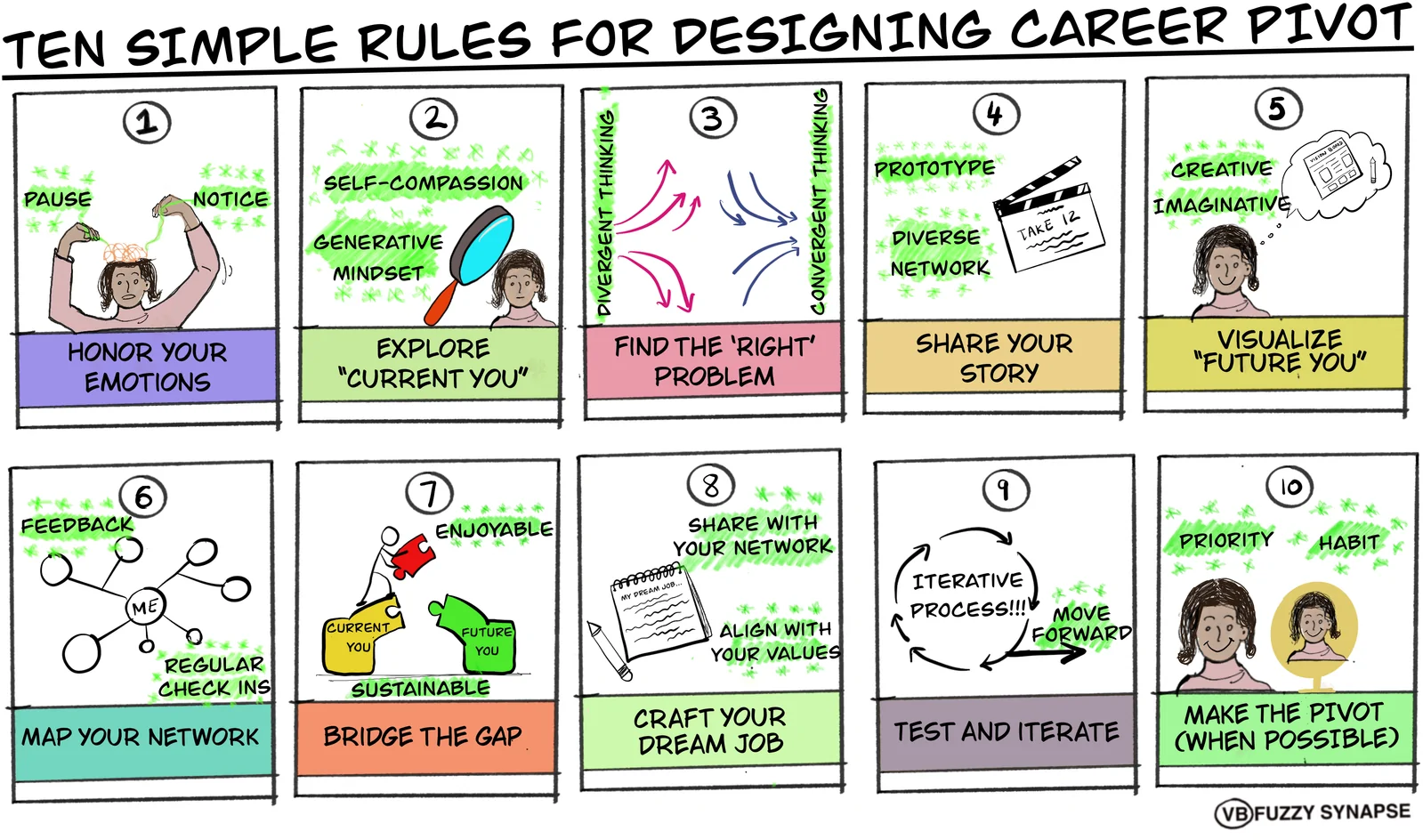
Illustration summarizing how to design your career pivot.

It is important to note that our pivot was gradual. We were under no immediate pressure like some readers who may be facing financial insecurity, visa hurdles, or caregiving responsibilities. Individuals with more time constraints may only afford to engage with a subset of the rules, and this is okay. These strategies will have value at different times of your life. Our goal is for this article to serve as a wholesome guide for those considering a career pivot at any stage and as a resource for mentors who support others in navigating career decisions.

## RULE 1: Honor your emotions

Career transitions can stir up a myriad of emotions that may conflict with one another [13–15]. While it might be unfamiliar and uncomfortable, it is important to notice the emotions you are feeling. At the beginning of our career pivots, we took a lot of time for introspection and noticed what emotions were surfacing. Typically, we view emotions as getting in the way of our tasks or goals, so we often attempt to push through them as quickly as possible. However, we decided to acknowledge our emotions and navigate them with empathy. This approach may feel dubious or challenging because, as scientists, we are trained to think objectively and analytically. However, emotions are great feedback that we can learn from and use to make better decisions as we navigate change [16]. In fact, using our emotions as a guide enabled us to be more analytical along our journeys. Being mindful of our emotional state allowed us to understand and prioritize our problems before they became overwhelming and gave us the capacity to solve them creatively.

You may be tempted to ignore your emotions during your career pivot, but please remain open and show yourself compassion by honoring your emotions at every step. Paying attention to our emotional state increases our self-awareness and well-being and can guide us effectively as we navigate the problem-finding and solving process inherent in change.

## RULE 2: Explore “Current you”

Sit with yourself and reflect on your personal and professional choices and immediate capacity. What emotions do you feel throughout the day when you are working? What challenges energize you? Which ones deplete your energy? Although taking inventory of your current situation is difficult, we invite you to nonjudgmentally explore what you are proud of and what you can learn from “current you”.

It’s okay if you don’t know the answers at this moment. Having these answers now would most likely mean you already engage in a practice where you check in and reflect on yourself periodically. Take time to think about these questions and write down your responses. Notice what patterns and additional questions emerge. Follow the trail and allow yourself to be in a generative mindset. In addition to answering questions like those above, we engaged in a values exploration exercise, which revealed what was important to us, as well as our negotiables and nonnegotiables. Have fun, be thoughtful!

By the time we engage in reflections like these, it is often due to a negative experience, which can sour the introspection and make self-compassion more difficult. We recommend making this activity a consistent, sustainable, and joyful practice that keeps you current on your values and priorities. You could start with one question per week if you aren’t used to engaging in self-reflection or if you are short on time or energy. We found that making this exercise into a practice, and thus detaching it from any single experience, enabled us to make the process more enjoyable. We couldn’t change the past, but design thinking taught us that pausing and reflecting can be instrumental in determining what decisions we want to make for our future.

Before moving on, pause to celebrate and be proud of what you’ve already accomplished. It takes a lot of energy and courage to self-reflect while holding a compassionate lens.

## RULE 3: Find the ‘right’ problem and set your intentions

Change gives us the opportunity to reimagine ourselves, but there are barriers along the way that need to be identified and overcome. Career transitions often reveal multiple challenges at once. You may feel the urge to choose all of them, but the goal is to reduce feelings of overwhelm. Reflection offers us time to identify the most important problems to focus on and consider actionable steps towards their resolution. For example, you may lack the time required to adapt your career materials for a new field. Or perhaps you may not even know how to adapt them. These are distinct problems that require distinct steps to solve them. So, which is your ‘right’ problem?

An effective activity each time you notice a problem is to articulate the problem and then successively ask “why” to uncover underlying problems, feelings, and thoughts [11]. You may identify many problems during this activity. Your goal here is to capture everything you are noticing and write it down. This can be done as a free-write, but if you prefer structure, you can use the Stop-Start-Continue concept [17] and organize your thoughts into what you want to stop, start, or continue doing. For example, something under “start” that you can’t start might be a problem to explore. You will use design thinking elements of switching between brainstorming problems (divergent thinking) and selecting one (convergent thinking) ‘right’ problem that, when addressed, can significantly move you forward in your pivot. Finding our ‘right’ problem enabled us to begin identifying the skills and opportunities needed to address the problem and a path forward to find joy and be in alignment with our value system.

Next, based on your ‘right’ problem, set an intention for how you want to navigate your career pivot. Intentions can provide direction on this journey and remind you to align your future behaviors to honor them. In our experience, this was an iterative process. We found it helpful to revisit our intentions periodically as new insights, opportunities, or constraints emerged. A lot can change in a short period of time.

## RULE 4: Share your story

Storytelling allows you to reflect and transform a collection of experiences into a clearer vision for your future [11]. As you construct and reconstruct narratives about your experiences, you may discover themes, motivations, and capabilities that were previously difficult to see. There is a need for storytelling at every stage of your career pivot, whether in elevator pitches and informational interviews, your resume and cover letter, or throughout the multi-stage interview process. Consider: Which stories do you want to tell? Is there a past project you are particularly proud of? A role that you assumed and found challenging, but quickly learned how to excel at it? Was there a tough decision you made that had no clear right or wrong answer, but you learned something valuable from it?

We invite you to prototype or test your stories and practice sharing them with people you trust, respect, and who can offer effective feedback. In design thinking, being aware of the language and narrative we use reveals mindsets, assumptions, and behavioral patterns that can hold us back or propel us forward [11]. Sharing your stories with multiple people with diverse backgrounds may enable you to identify strengths, skills, and values that weren’t previously clear to you. The process can refine your stories into effective, compelling narratives that will strengthen your position during your career pivot. In our experience, it was important for us to share our stories with multiple people across industries. They were able to offer distinct interpretations and highlight different aspects of what we shared and some of these learnings directly influenced the next steps we decided to take in our pivots. We practiced so many iterations of our stories that, by the time we interviewed, we were comfortable and confident in sharing our narratives.

Sharing your stories with others can be challenging. This is yet another opportunity for you to practice pausing and noticing. Acknowledge your emotions and thoughts, as well as notice the reactions of your listeners. Importantly, the purpose of this exercise is not to seek permission or validation. While feedback from others can be valuable, remember that you are the ultimate author of your own story. Others may not fully understand your envisioned future, and that is okay. Use feedback as information to consider rather than as approval that must be obtained before moving forward.

## RULE 5: Visualize “Future you”

While previous rules guided you in distinct ways to reflect on and better understand your past and present, this rule invites you to envision different possible versions of the “future you.” Visualize a job that would support you holistically and bring you joy, awe, or excitement. What tasks are you completing? What emotions are you feeling throughout your workday? What strengths are being employed? What challenges are you enjoying? We suggest you go somewhere that you know activates a creative, imaginative, yet perhaps calm headspace. This may be on a hike, in your garden, during asana, or during a seated meditation.

Now, take what you visualized as your “future you” and bring it into physical form. Start by briefly writing notes on what you visualized and how you answered the questions above. Then, turn your notes into a creative activity. For example, if you love writing, expand on your notes and journal these new stories you are creating for yourself. If you are more visual, consider creating a vision board [18].

You don’t have to stop here. You can repeat this practice and create multiple stories for yourself. We found that this is an opportunity to explore multiple “future you” versions whose jobs align with your values, support your well-being, and inspire you to maintain your priorities. Through iteration, ideas will develop over time and reveal potential next steps. Recall Rule 4 and share your stories with others. This may seem silly since these are imagined futures, but as you share them with others, you may notice which ones feel a little more ‘right’ for you.

## RULE 6: Map your network and build meaningful connections

Networking is an important skill we all know of but still find difficult to build and use when needed. We initially dreaded even thinking about it, but then we realized it was just connecting with others. It is an intentional act that can bring together people with diverse perspectives and backgrounds to create a community that provides feedback, accountability, and a supportive environment for finding solutions. Networking is especially important in tough job markets, when so many highly skilled individuals are seeking new opportunities simultaneously. It is the folks in your network who will be instrumental in facilitating connections. So, we suggest doing an activity called ‘Map your network’ [11].

With you at the center, draw any connections that can contribute to your personal, professional, and career advancement. Remember to include your casual connections! Your loose acquaintances can connect you to networks beyond your circle, enabling you access to ideas, information, and opportunities you would otherwise miss [19,20]. Who can you contact to learn certain skills and competencies? Who has experience strategizing career pivots? Who can offer effective feedback on your resume and interview practice? The goal is to identify your needs and cultivate a network that can meet them. Taking inventory of your current relationships will allow you to identify the strengths and limitations of your current network. Is your network currently diverse enough to fulfill the roles necessary for your success? If not, who is missing? The more diverse your network is, especially in career pivots, the more it will benefit you. Building a diverse network requires that you strengthen and maintain current relationships as well as establish new ones. Over time, with effort, you’ll be able to move these people closer to you in the center [21]. You will return to this rule as you work through bridging the current and future versions of yourself in the next rule - with each new gap, you may consider who in your network or your network’s network can shrink this gap.

You might feel shy, overwhelmed, or worried about making the wrong impression when connecting with others during your career pivot. We certainly did! But we encourage you to push yourself to reach out anyway. For us, it got easier with time, and dare we say, enjoyable. We were even able to move beyond networking and build meaningful connections with others who could assist in our career pivots!

## RULE 7: Bridge the gap between “Current” and “Future” you

Recall “current you” (Rule 2) and “future you” (Rule 5) and think about the gaps that are keeping these two versions of yourself separate. Strategically order these gaps based on priority and/or ease. We suggest that you pick one or two, as it might not be feasible to address all gaps due to time, budget, or other factors. How can you upskill yourself to address these gaps? Which gaps can you address independently, and which ones require support from others? Would informational interviews provide enough insight into the typical day in the life of someone whose job intrigues you, or is job shadowing an option?

Leverage your network and think about which people can assist you in bridging these gaps. Speaking with others will most likely enable you to identify gaps as well. If you notice that you don’t currently have anyone in your network with the expertise relevant to address specific gaps, you can explore your network’s network.

Once you have a large list of actions and ideas of how to bridge the gaps, choose one that you can commit to today and that will bring you one step closer to what you want and where you want to be. Each gap will require a different level of effort and support to shrink or close it, so pause and notice: what do you have the capacity to take on today? Some of these actions might seem daunting, but you don’t have to do them alone. We tapped our friends, family, and other folks in our networks—being in community created the momentum and support we needed to make this process sustainable, enjoyable, and productive.

## RULE 8: Craft your dream job

To make your “future you” more tangible, you can prototype your ideas! This rule focuses on turning what you previously visualized into experiences that you can explore and evaluate. We offer two prototyping activities that benefited us during our journeys, depending on your capacity and preference: **create a job description** [22] and/or engage in a **career experiment** [23].

The first is crafting a **job description.** It doesn’t need to be a formal, highly structured document, but it can resemble job descriptions found in the field you want to pivot into. We recommend that you look up posted job descriptions and reflect on what you like or don’t like about each one, remembering to notice your thoughts and emotions. Now you can create your own document. Think back to Rule 5 and the list of things your future self wants to do because of this pivot. Brainstorm 5–10 ideas for different opportunities that would allow you to pursue the things you want. What would you like to see highlighted in each of these sections? What duties and responsibilities do you want to engage in on a daily or weekly basis? Consider how important they are to you and how frequently you would want to engage in them during the work week. What skills and qualifications do you have that support each duty and responsibility you listed? What do you want the company’s values or mission to be? What other details do you want to add to this job description that will actualize your “future you”? Noticing how much you know and don’t yet know will reveal additional gaps and perhaps more bridging actions.

The second is crafting **career experiments**. You can examine aspects of jobs you have visualized for your future self and identify opportunities to test them. For example, you might complete an internship, volunteer for a cross-functional project, or pursue an independent project that allows you to explore skills and responsibilities associated with your pivot. Remain open to opportunities that aren’t necessarily on your radar, as they may reveal unexpected career possibilities. You may want to ask yourself: What is an aspect of a job you’ve visualized and crafted for “future you” that excites you and honors your intention? Given your current personal and professional responsibilities and general bandwidth, what is a realistic, sustainable action you can commit to? Be detailed and specific so that you are clear on what you will test. Do you want your commitment to be daily, weekly, or monthly? When do you want your experiment to end?

As design thinking encourages, we invite you to test possible futures through low-risk prototyping before making major career decisions. These prototype activities are opportunities for you to coalesce what you’ve learned through reflection and transform your vision into a tangible product that can be shared with others and tested.

## RULE 9: Test and iterate

When you have a plan, it is time to test it out and share it with others! As design thinking teaches, these prototypes should be tested and iterated through so that we learn and move forward. While we were postdocs exploring our pivots, we used our network to share our career experiment/job description and brainstormed opportunities within our network to pursue alongside our postdoc responsibilities. We both found opportunities that allowed us to explore roles we envisioned in versions of our “future you”. We learned what we liked and disliked, and we reflected throughout about whether these positions aligned with our values. It is natural for us to be attached to ideas or prototypes we’ve invested time and energy into developing, but design thinking taught us that it is important to test and iterate while remaining open and curious!

In our experience, sharing prototypes with others yields effective feedback, more creative ideas, and clarity about a path forward. Through multiple iterations and proper reflection, you will become more informed and better refine your “future you”. This is why we recommend sharing your prototypes early and often with your diverse network. It might be scary, but bringing people into the fold can facilitate you finding your dream job, or even having one created for you!

## RULE 10: Make the pivot (when possible)

After spending so much time exploring careers or being on the job market, any job opportunity might seem like a great one. While you may be eager to jump at it, we encourage you to pause and remember why you began this job search in the first place. Will this decision address your ‘right’ problem and honor your intention? If you have the privilege of time, financial stability, and emotional support, go back to your values, the purpose you want to serve, and your job description to see how much this job opportunity aligns with what you visualized for your future. If there is extensive misalignment, then you can feel emboldened to continue looking.

If you are currently in an unstable situation and cannot afford to wait for more opportunities to come along, then you’ll most likely need to accept the offer, whether it honors your nonnegotiables or not. However, accepting a job doesn’t mean that your search for your imagined future must end. You now have prototypes that you can update to reflect the ‘current you’, so you don’t have to start from scratch next time! An empowering reframe can be that you are engaging in a series of smaller pivots or at least gaining skills and experiences that place you one step closer to achieving the ultimate goal of having your dream career.

Even when you do find a job that brings you joy, honors your nonnegotiables, and nearly checks off everything you listed on your job description, your search for a better future should continue. As you change over time, your values, priorities, and interests will follow. We encourage you to create intentional time for introspection. By making reflection your priority and a habit, you’ll always know when to make your next move.

## Conclusion

Creating new futures can be a daunting task. Grounded in design thinking and with emphasis on emotional awareness and well-being, the ten simple rules listed above offer opportunities for you to reframe career pivot challenges and address them creatively. With ongoing self-awareness and insight, you’ll know whether to stay or when it’s time to start your next pivot. We hope that by applying these design thinking-inspired rules, the career pivot process becomes empowering, sustainable, and enjoyable for everyone.
